# Lipodystrophic laminopathies: Diagnostic clues

**DOI:** 10.1080/19491034.2018.1454167

**Published:** 2018-04-16

**Authors:** Cristina Guillín-Amarelle, Antía Fernández-Pombo, Sofía Sánchez-Iglesias, David Araújo-Vilar

**Affiliations:** UETeM-Molecular Pathology Group, Department of Medicine, IDIS-CIMUS, University of Santiago de Compostela, Spain

**Keywords:** diagnosis, laminopathies, LMNA, progeria, type 2 familial partial lipodystrophy

## Abstract

The nuclear lamina is a complex reticular structure that covers the inner face of the nucleus membrane in metazoan cells. It is mainly formed by intermediate filaments called lamins, and exerts essential functions to maintain the cellular viability. Lamin A/C provides mechanical steadiness to the nucleus and regulates genetic machinery. Laminopathies are tissue-specific or systemic disorders caused by variants in *LMNA* gene (primary laminopathies) or in other genes encoding proteins which are playing some role in prelamin A maturation or in lamin A/C function (secondary laminopathies). Those disorders in which adipose tissue is affected are called laminopathic lipodystrophies and include type 2 familial partial lipodystrophy and certain premature aging syndromes. This work summarizes the main clinical features of these syndromes, their associated comorbidities and the clues for the differential diagnosis with other lipodystrophic disorders.

## Introduction

Lamins are 60–70 kDa proteins belonging to type V intermediate filaments [1,2]. They are assembled into increasingly complex associations of paracrystalline structures to build the nuclear lamina, a meshwork covering the inner face of the nuclear membrane [3]. Little amounts of lamins also localize in the nucleoplasm [4]. The nuclear lamina is reversibly disassembled during mitosis [5], and plays a decisive role in functional, structural and three-dimensional organization of chromatin throughout cell differentiation [2,3]. Lamins exert transcendental biological functions: maintenance of nuclear structure, organization of cytoskeleton, regulation of gene transcription, cell cycle and apoptosis; differentiation of stem cells and cellular migration [6–9]. Nuclear lamina could be considered as a messaging centre for the organization and distribution of information in cells, through its interactions with chromatin, transcription factors, nuclear envelope proteins, nuclear pore complexes and cytoplasmic structures as microtubules and other intermediate filaments [6].

Type A lamins exhibit a high degree of evolutionary conservation, and are similarly expressed in most tissues, except in the central nervous system, where C-type lamin abounds in the brain, while A-type lamin and its precursor (prelamin A) are expressed in endothelial and meningeal cells, but not in the neurons [10,11]. Lamins integrity is critical for human health. Damages in nuclear lamina lead to over 17 diseases, collectively termed laminopathies. B-type lamins are essential for DNA replication, so their mutations are often incompatible with life [12]. Most laminopathies come from changes in A-type lamins, and include myopathies (striated muscle), cardiomyopathies, neuropathies (peripheral nerves), lipodystrophies (fat) and premature aging syndromes (systemic laminopathies) [7,13].

Laminopathies are considered rare diseases because of their low prevalence, although no specific epidemiological studies have been conducted so far [14]. More than 300 pathogenic variants (usually missense) have been reported in *LMNA* gene, characterized by a broad and heterogeneous spectrum of clinical manifestations. For more detailed information, see Leiden Open Variation Database (http://www.dmd.nl/nmdb/variants.php?action = search_unique&select_db = LMNA). Primary laminopathies include those diseases caused by variants in *LMNA*, whilst secondary laminopathies are caused by variants in other genes encoding proteins which are playing some role in prelamin A maturation (*ZMPSTE24*) or in lamin A/C function (*BANF1*).

Overlapping syndromes are even odder, with a variable involvement of mesenchymal tissues (fat, skeletal and cardiac muscle, and bones), peripheral nerves and premature ageing stigmata [15].

Mutations causing myopathy are distributed all along the *LMNA* gene, and are the most common (60% of laminopathies), whilst the 75% of the mutations causing lipodystrophies affect the IgG-like domain [16].

Laminopathy lipodystrophies encompass type 2 familial partial lipodystrophy (Dunnigan disease) and certain early aging syndromes.

A written consent was obtained from the patients for the publication of their photographs.

## Dunnigan disease

Type 2 familial partial lipodystrophy (FPLD2; MIM: #151660) or Dunnigan disease results from heterozygous or compound heterozygous variants affecting mainly exons 8 and 11 of *LMNA* gene (1q21–22, NC_000001.11), although variants in other exons have been reported [17,18]. The classic phenotype comes from variants in exon 8, particularly the p.R482W/Q one, which is currently related with 80% of FPLD2 cases, and produces the more severe lipodystrophic phenotype [19–23].

**Figure 1.**
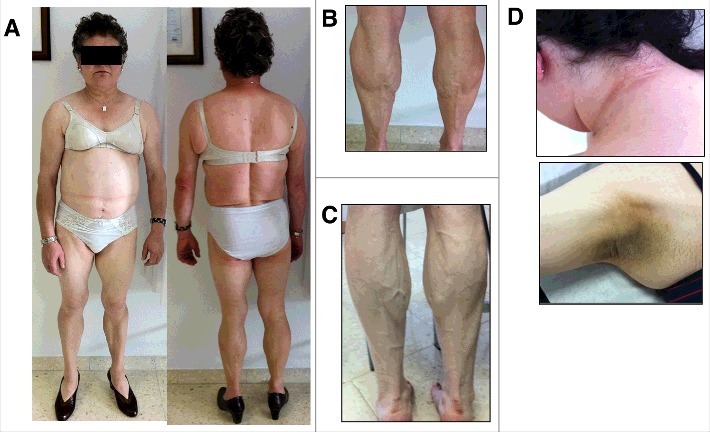
Type 2 familial partial lipodystrophy. A: typical phenotype in a 60 years old woman carrying the p. R482Q *LMNA* variant. B: Calf muscular hypertrophy. C: Phlebomegaly. D: Acanthosis nigricans in neck and axillae.

**Figure 2.**
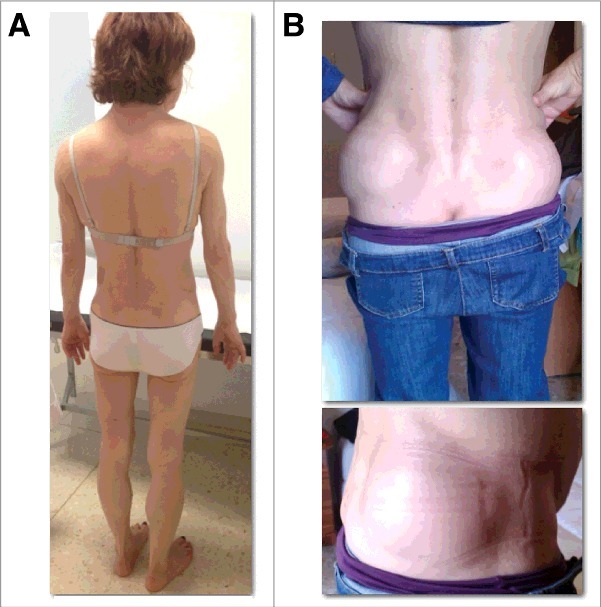
58 year-old women with FPLD2 due to p.N466D *LMNA* variant (A). One year later 2 large non-encapsulated lipomas appeared over the iliac crests (B).

The hallmark of FPLD2 is the lost of fat starting around puberty in women affecting limbs, trunk, hips and buttocks, and fat accumulation in face, neck, axillae, dorsal region, labia majora and visceral region (Figure 1A) [24]. The well defined and increased musculature (Figure 1B), plus this particular fat distribution, confer these women an android appearance [14,25]. Phlebomegaly is frequent in upper and lower limbs (Figure 1C). The hands use to be wide with short fingers. Acanthosis nigricans in neck and axillae and achocordons, as insulin resistance stigmata, are not unfrequent (Figure 1D). In men, this pattern of lost of fat appears later and it is less evident [26]. In fact, affected men are usually diagnosed from their female relatives. The presence of subcutaneous lipomas, not present in all patients, could make the clinician suspect of type 2 FPLD in the context of a FPLD phenotype (Figure 2) [23].

These patients have metabolic, cardiovascular, hepatic and pancreatic comorbidities. Insulin resis-tance is frequent from youth, the same as hypertriglyceridemia and low HDL-cholesterol [27]. In some cases non-ketotic diabetes mellitus appear during adulthood as acute pancreatitis as a consequence of severe hypertriglyceridemia, however, in our experience, life style, particularly diet, is conditioning the apparition of these entities. Hepatic steatosis is frequent, generally associated with high aminotransferase plasma levels, being uncommon hepatic cirrhosis [28]. Affected women present with gynaecological disorders as polycystic ovarian syndrome, gestational diabetes, miscarriage and stillbirth [29].

The cardiovascular spectrum of this lipodystrophy is wide including early atherosclerotic cardiovascular diseases, rhythm disturbances, valvulopathies or hypertrophic cardiomyopathy [27,30–33].

Strikingly, the prevalence of metabolic disturbances and atherosclerotic vascular disease in more frequent in women than in men [34]; on the other hand, it has been reported recently an anticipation phenomenon in relation with metabolic complications of Dunnigan disease [35].

Serum leptin levels tend to be low in familial partial lipodystrophies, although no specific threshold has been defined as diagnostic criteria [14]. In general, Dunnigan treatment includes that of the associated comorbidities, according to the international clinical guidelines [36–38].

It is important to highlight a growing body of data supporting the pivotal role of lamins in metabolic syndrome pathogenesis. Non classical mutations have been associated with abdominal obesity but moderate or absent distal lipoatrophy. This particular manifestation of metabolic syndrome has been named the “metabolic laminopathy” [39].

### Differential diagnosis among familial partial lipodystrophies

FPLD encompasses eight Mendelian disorders involving abnormal body fat distribution and insulin resistance (Table 1) [40]. They share a cushingoid appearance, resulting from the loss of subcutaneous fat in the limbs and gluteal region since childhood or puberty, associated with a variable excess of adipose tissue in the face, neck and intra-abdominal region.

**Table 1.** Characteristics and differential diagnosis of partial lipodystrophies.

DISEASE	GEN PROTEIN FUNCTION	PHENOTYPE
FPLD1 [41, 42] (Köbberling)	UNKNOWN	Childhood onset. Worsens in menopause.
		Lower limb subcutaneous lipodystrophy, variable in the upper limbs.
		Fat accumulation in the abdomen (subcutaneous and intra-abdominal), cervico-thoracic, face.
		Insulin resistance and metabolic syndrome.
FPLD2 [14, 17–39] (Dunnigan)	*LMNA* (heterozygous)	Pubertal onset.
	Lamin A/C	Subcutaneous fat loss in the lower limbs, upper limbs and abdomen.
	Nuclear lamina (see text)	Fat excess in the face, neck, axillae, labia majora, intra-abdominal region. Insulin resistance and metabolic syndrome.
		Cardiac abnormalities may be present.
FPLD3 [43, 46	*PPARG* (heterozygous)	Adult onset.
	Peroxisome Proliferator Activated Receptor ϵ	Less severe fat loss.
	Adipogenesis	No fat accumulation in the face and neck.
		Severe metabolic complications.
		Severe hypertension.
FPLD4 [47, 48]	*PLIN1* (heterozygous)	Childhood onset.
	Perilipin 1	Limb lipodystrophy.
	Lipid droplet	Facial / neck fat accumulation may be present or absent.
		Insulin resistance and metabolic syndrome.
FPLD5 [50]	*CIDEC* (homozygous)	Childhood onset.
	Cell Death Inducing DFFA Like Effector C	Limb and abdominal subcutaneous lipodystrophy, no abnormal fat depots.
	Preadipocyte differentiation, lipid droplet	Marked hypermuscular appearance.
		Ketosic diabetes, albuminuria.
FPLD6 [49, 51	*LIPE* (homozygous)	Adult onset.
	Hormono sensitive lipase	Limb lipodystrophy.
	Regulation of lipolysis	Fat accumulation in a “Cushing” fashion including back.
		Insulin resistance and metabolic syndrome.
		Muscular dystrophy with high CPK levels.
LCCNS [53]	*CAV1* (heterozygous)	28 yr old woman.
	Caveolin 1	Atypical lipodystrophy of the upper body.
	Caveolae formation	Severe hyperlipemia, recurrent pancreatitis.
		Congenital cataracts.
		Neurodegeneration syndrome.
FPLD [52]	*AKT2* (heterozygous)	Adulthood onset.
	AKT Serine/Threonine Kinase 2	Partial lipodystrophy.
	Insulin signalling pathways	Marked hyperinsulinemia, diabetes around 30 years of age.

LCCNS:Lipodystrophy, partial, with congenital cataracts and neurodegeneration.

**Figure 3.**
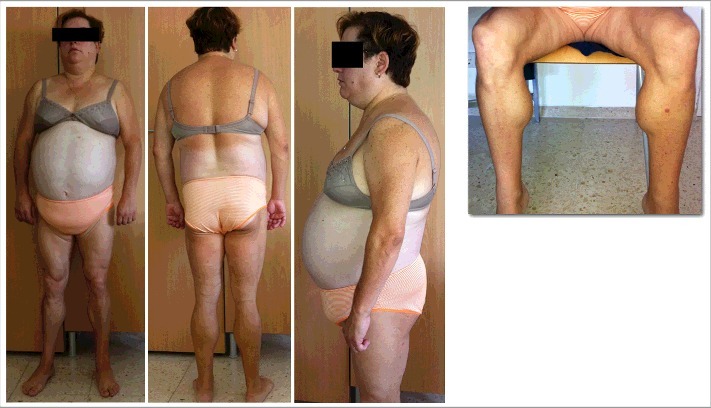
33 years old woman with type 1 familial partial lipodystrophy. No pathogenic variants were found in *AGPTA2, AKT2, BANF1, BLM, BSCL2, CAV1, CIDED, ERCC6, ERCC8, FBN1, KCNJ6, LIPE, LMNA, PCYT1A, PIK3R1, PLIN1, POLD1, PPARG, PSMB8, PTRF, WRN, SPRTN* and *ZMPSTE24* genes.

Type 1 FPLD or Köbberling syndrome (MIM: %608600) is an inherited variety of FPLD, although specific genes are unknown, and a polygenic or oligogenic pathogenesis has been suggested [41,42]. Phenotype starts at childhood, and worsens with menopause and weight gain. Insulin-resistant diabetes mellitus, hypertriglyceridemia and cardiovascular disease are frequent. Lipoatrophy is present in lower limbs and occasionally in upper limbs. Patients are frequent obese and have a remarkable accumulation of abdominal fat (including subcutaneous fat), so they are usually diagnosed with android obesity (Figure 3) [41].

Variants in *PPARG* gene (3p25.2; peroxisome proliferator activated receptor gamma) are responsible for type 3 FPLD (MIM: #604367), an autosomal dominant disease. Despite the extreme severity of this lipodystrophy, fat loss is less striking and starts later, during the second decade of life. Usually, there is no fat accumulation in the face and neck. Clinical manifestations are more severe than in Dunnigan disease. Insulin resistance is extreme, with early onset of diabetes and pancreatitis. Severe and poorly controlled hypertension can also occur and, in the case of pregnancy, eclampsia [43–46].

Type 4 FPLD (MIM: #613877) is due to heterozygous variants in *PLIN1* (15q26.1; perilipin 1), starting at childhood. Like in FPLD2, affected patients have lower limbs lipoatrophy with fat excess in face and neck, calf hypertrophy, hypoleptinemia, severe dyslipidemia, insulin resistant diabetes, and high blood pressure. BMI is usually less than 30 [47,48].

FPLD types 5 and 6 are rare recessive conditions [49–51]. Type 5 FPLD (MIM: #615238) is due to an homozygous variant in *CIDEC* gene (3p25.3, Cell Death Inducing DFFA Like Effector C). Only a patient with this disorder has been reported with classical FPLD features since early childhood. Particular features of this lipodystrophy are muscular hypertrophy but no accumulation of adipose tissue, ketosic diabetes and albuminuria [50].

Type 6 FPLD (MIM: #615980) is an adulthood onset lipodystrophy resulting from variants in *LIPE* (19q13.2, lipase E, hormone sensitive type) [49]. Patients suffer from partial lipodystrophy with accumulation of fat in the neck, axillae, back, and supraclavicular area. A particular trait is the presence of muscular dystrophy with high levels of creatine kinase. Very rare forms of FPLD have been linked to mutations in *AKT2* (19q13, AKT Serine/Threonine Kinase 2) and *CAV1* (MIM: **#**606721, 7q31.1, Caveolin-1), see [Table 1. 52,53]

## Overlapping laminopathies with lipodystrophy

Most fascinating traits of laminopathies are the complex genotype-phenotype associations and their clinical heterogeneity [54]. Thus, the same variant can lead to different phenotypes, and a similar phenotype can in turn come from different variants. It is possible that some of these variants in *LMNA* may modulate the expressiveness of others, even in distinct genes. So, some years ago Savage et al [55]. published the case of a female with partial lipodystrophy, who carried two missense variants in *LMNA*: c.1748C>T (p.S583L) in exon 11, inherited from her father; and c.1583C>T (p.T528M) in exon 9, inherited from her mother. Relatives with only one of the variants were not lipodystrophic, while those with both of them had a typical Dunnigan phenotype. A couple of years later, Verstraeten et al [56]. reported a male with a progeroid syndrome, who carried a pair of variants in *LMNA*: c.1619T>C (p.M540T) in exon 10, inherited from his mother, and again the c.1583C>T (p.T528M) in exon 9, inherited from his father. Parents were apparently healthy, although their cells showed nuclear abnormalities similar to those of laminopathies.

Laminopathies clinical heterogeneity is also expressed in overlapping syndromes, characterized by the coexistence of lipodystrophy with myopathy, neuropathy and/or premature ageing stigmata, giving rise to the concept of a multisystem dystrophy syndrome [57]. Environmental factors, other modifier genes or epigenetic modifications could explain these particular diseases. Specific mutations are also determinants in the phenotypic heterogeneity. In fact, the same mutation can cause different diseases, and similar diseases can arise from different mutations.

Associations between cardiomyopathy and myopathy are frequent, but concurrence of lipodystrophy, cardiomyopathy and myopathy is not so unusual [58]. Several *LMNA*-associated complex phenotypes have been reported, including muscular dystrophy, lipodystrophy, and cardiac rhythm disturbances related to a R527P variant; or FPLD, early heart failure, first-degree atrioventricular block, and late proximal muscle weakness, due to a R28W variant [57].

Heterozygous missense mutations all along the *LMNA* gene cause the majority of overlapping laminopathies [15]. Although deletions are rare (6% of known mutations), the sporadic heterozygous change c.1001_1003delGCC (p.Ser334del) affecting exon 6 also illustrates the broad clinical spectrum of laminopathies, not always easy to discern [59]. It manifests with severe heart failure overlapping with lipodystrophy.

Some other examples are listed below: R545H causes an association of FPLD, proximal myopathy and cardiomyopathy [60–62]; T655fsX49, which was found in the Reunion Islands, is of particular interest [32]. Homozygous fibroblasts carrying this mutation present higher amounts of prelamin A than heterozygous, which translates into higher oxidative and senescence rates and, at the clinical level, into a more severe overlapping syndrome of partial lipodystrophy, atherosclerotic disease and cardiomyopathy with conduction abnormalities and ventricular dysfunction.

In summary, overlapping syndromes deepen the need to actively seek the presence of *LMNA* mutations in subjects with clinical features of partial lipodystrophy, cardiac abnormalities, or certain types of myopathy, in order to prevent, as far as possible, their potentially lethal consequences.

## Hutchinson-gilford progeria syndrome

Progeroid syndromes are characterized by the presence of general premature ageing stigmata, as alopecia, graying, osteoporosis, joint contractures, a varying degree of lipodystrophy, loss of muscle mass or senile skin changes, among others [63–65]. Interestingly, nor intellectual impairment neither progressive dementias are typical manifestations of most of the progerias [66].

**Figure 4.**
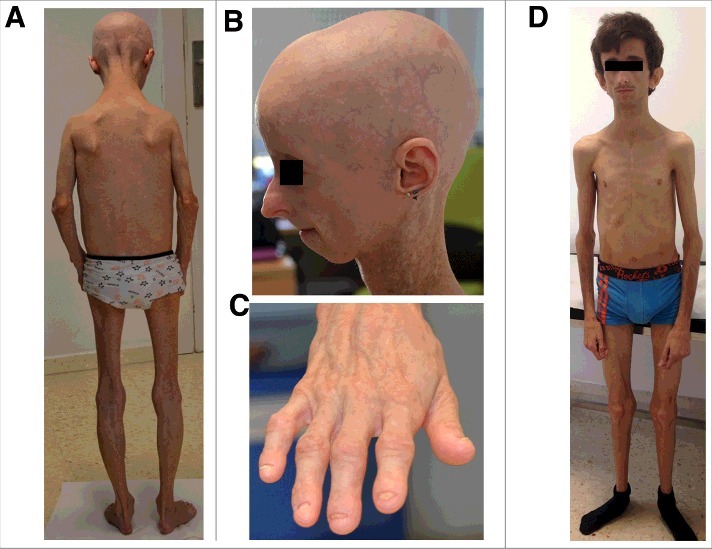
16 years old woman with HGPS due to p.G608G *LMNA* variant. A. Generalized lipodystrophy. B: Typical facial features of HGPS (alopecia, beaked nose, micrognathia) and leucomelanodermic lesions in neck. C. Dystrophic nails. D: Atypical progeria syndrome in a 16 year old man due to *de novo* p.T10I *LMNA* variant.

Phenotypical hallmarks of Hutchinson-Gilford Progeria Syndrome (HGPS) (MIM: #176670) are similar regardless of gender or ethnicity [67]. Patients are normal at birth. Particular physical appearance starts to be evident at 18–24 months of age, and includes a broad range of signs and symptoms [68,69] (Figure 4):failure to thrive and growth retardation, short stature, low body weight, incomplete sexual development (prepuberal); disproportionally large head with high arched palate, beaked nose, micrognathia, circumoral cyanosis, mandibular osteolysis and dental crowding; generalized lipodystrophy preserving intraabdominal fat; acroosteolysis, osteopenia and osteoporosis, reduced muscle mass and articular stiffness with restricted mobility. Skin becomes thinner and sclerotic, plenty of senile spots, with prominent vasculature. Additional clinical features are conductive or high-frequency sensorineural hearing loss, early alopecia with prominent scalp veins and nail dystrophy. Malignant neoplasms are not typical in HGPS.

Biochemical tests can show prolonged prothrombin times, elevated platelet counts and elevated serum phosphorus levels. Fasting insulin values can be elevated, and sometimes accompanied by non-ketotic diabetes and hypertriglyceridemia [70].

HGPS patients have evidence of adventitial thickening, and at the same time low vascular compliance. They suffer from high blood pressure, leading to biventricular hypertrophy and biatrial enlargement [70]. Cardiovascular disease (stroke, myocardial infarction) causes early death, with an average life expectancy of 13.4 years old (7–27.5) [69,70].

## Néstor guillermo progeria syndrome

This progeria (MIM: #614008) got its name from the 31 and 24 yr old reported patients, Néstor and Guillermo, who came from two unrelated, consanguineous, Spanish families. The disorder is defined as a secondary laminopathy. It arises from a homozygous missense variant in *BANF1* (11q13.1), the gene coding for the Barrier to Autointegration Factor (BAF), a protein which mediates interactions between lamins and chromatin throughout the cell cycle [71].

Affected patients presented with normal development until the age of two years. From that moment, they showed a failure to thrive and a peculiar appearance including ageing features: micrognathia, convex nasal ridge, proptosis, atrophic skin with senile skin spots, generalized lipoatrophy giving a prominent appearance to superficial veins. They suffered from osteoporosis, marked scoliosis since 18 yr old and severe osteolysis of mandible, maxilla, clavicles, ribs and distal phalanges. Contrary to what happens in HGPS, both patients were taller (145 cm) and preserved eyebrow, eyelashes, and the scalp hair at least to the age of 12 yr old. However, the most important difference between these disorders lies in two clue points: much greater life expectancy and absence of atherosclerosis, and metabolic syndrome in Néstor-Guillermo Progeria Syndrome. In fact, some call it “the chronic progeria” because of the more indolent clinical course and longer survival. By counterpart, patients showed secondary pulmonary hypertension and a severe restrictive spirometry pattern with biatrial enlargement [71,72].

## Mandibuloacral dysplasia with type a lipodystrophy

Mandibuloacral dysplasia type A (MADA, MIM: #248370) is a very rare autosomal recessive disorder (homozygous or compound heterozygous) due to variants in *LMNA*. The most frequent variant is the R527H, which alters the recognition site for *ZMPSTE24*, leading to prelamin A accumulation [73].

MADA is identified between childhood and puberty (average 5 yr) by short stature (some cases with accelerated aging) and particular phenotypic features: pointed noise, high arched palate, sparse scalp hair, craniofacial anomalies as mandibular and dysplasia with hypoplastic mandible and dental crowding, clavicular dysplasia, osteoporosis, progressive osteolysis of distal bones, persistently wide cranial sutures, multiple wormian bones, anomalous skin pigmentation and premature aging features as stiff joints. It could be said the disease behaves as a diffuse affection of the connective tissue. Interestingly, in MAD osteolysis is not confined to hands and clavicles, but with years, it may extend to other skeletal regions, as elbows [73-76].

Lipodystrophy pattern is partial, and associated with extreme insulin resistance and marked hypermetabolism [77]. Patients show normal glucose tolerance but fasting and postprandial hyperinsulinemia and hypertriglyceridemia with low HDL levels can be present. Moreover, a premature adrenal cortical dysfunction has been seen in some cases, as occurs in normal ageing (Table 2) [78].

**Table 2.** Characteristics and differential diagnosis of laminopathic progeroid syndromes.

DISEASE	GENE	ONSET (YR)	LIFESPAN	LPD	PARTICULAR COMPLICATIONS	PECULIAR TRAITS
APS[84–90]	*LMNA*	4–17	?	GENERALIZED/PARTIAL	Valvulopathy Cardiomyopathy	No acro-osteolysis, no clavicular resorption
HGPS[63–70]	*LMNA*	1–2	13	GENERALIZED	Atherosclerosis, CV disease, metabolic syndrome	Global alopecia, no eyelashes.
MADA[73–78]	*LMNA*	2–4	>15	PARTIAL	Metabolic syndrome	Delayed closure of fontanels, wormian bones.
			(2 cases >40)			
MADB[79–83]	*ZMPSTE24*	perinatal	>20	GENERALIZED	Metabolic syndrome	Delayed closure of fontanels, wormian bones, SC nodules
NGPS[71,72]	*BANF1*		>30	GENERALIZED	Pulmonary hypertension, respiratory restrictive pattern	Profound skeletal abnormalities
			“THE CHRONIC PROGERIA”			

APS: Atypical progeria syndrome; HGPS: Hutchinson-Gilford Progeria Syndrome; MADA: Type A Madibuloacral dysplasia; MADB: Type B Madibuloacral dysplasia; NGPS: Nestor-Guillermo Progeria Syndrome.

## Mandibuloacral dysplasia with type b lipodystrophy

MADB (MIM: #608612) is a secondary laminopathy, resulting from homozygous variants in zinc metalloprotease *ZMPSTE24* (1p34.2) [79]. Phenotype appears at birth, with postnatal growth retardation and difficult to feed [80]. In fact, premature birth is not rare. Children have small chin and pinched nose, small mouth, dental overcrowding and retrognathia. They suffer from contractures because of tighten and tense skin. Typical features are pigmented cutaneous spots, delayed closure of fontanels, persistent wormian bones, small and hypoplastic clavicles, distal osteolysis and other ageing stigmata as neurosensorial deafness or hair loss. A differential factor of this progeroid syndrome is the presence of sclerotic and calcified subcutaneous nodules, lack of acanthosis nigricans, renal disease (glomerulopathy) and a generalized pattern of lipodystrophy (Table 2) [79,81,82]. As in MADA, glucose tolerance is normal, but there is hyperinsulinism in fasting and postprandial states, hypertriglyceridemia and low HDL levels [83].

## Atypical progeroid syndromes

Atypical progeroid syndromes (APS) constitute a small set of disorders due to heterozygous missense mutations in *LMNA*, with a slightly delayed onset of clinical manifestations when comparing with HGPS and MAD (Figure 4D) [84]. In the same way patients seems to live longer, even more than 50 yr old [85].

Clinically they are marked heterogeneous, but share several common characteristics with the rest of premature ageing syndromes, as graying of hair, neurosensorial deafness in some cases, sclerotic skin with mottling, joint stiffness, alopecia (sometimes slight or absent), small mandible, abnormal teeth implantation with overcrowding, high arched palate or beaked nose [86,87]. However, unlike patients with MAD or HGPS, in APS acroosteolysis is absent or mild, affecting only distal phalanges, and the same can be said of the clavicular hypoplasia (Table 2) [84]. Interestingly, despite normal menstrual cycles, poorly developed breasts are common in women with APS. Premature ovarian failure has been reported only in a few cases [84].

At the cardiovascular level, severe abnormalities in cardiac valves are common, including mitral, aortic and sometimes tricuspid, regurgitation, as well as aortic stenosis. Patients often undergo cardiac transplantation because of dilated cardiomyopathy [84]. As regards to the type of lipodystrophy in APS, it can be generalized (with or without excess of visceral fat) or partial, and be accompanied by diabetes, hypertriglyceridemia and fatty liver disease with hepatomegaly. Usually, metabolic alterations are worse than those seen in HGPS or MAD and, strikingly, acanthosis nigricans is absent [86,88].

A premature aging syndrome has recently been reported associated to variants in codon 55 (exon 1) in the *LMNA* gene [89]. The clinical picture of this LMNA-Associated Atypical Neonatal Progeria, reported in three children, recapitulate those of patients with HGPS and MAD. However, the symptoms appear early in life, lipodystrophy can be generalized or partial, and the prognosis is poor in relation to retrognatia-associated obstructive apneas and stroke.

Finally, Dilated Cardiomyopathy with Hypergonadotropic Hypogonadism is an atypical, late-onset, form of HGPS due to missense variants in the *LMNA* gene (A57P and L59R) (MIM: #212112) [90]. It is characterized by the presence of dilated cardiomyopathy, early ovarian failure, generalized lipodystrophy associated with insulin resistance, and progressive facial and skeletal changes (clavicular hypoplasia, low bone density, acrogeric appearance). Unlike the classical form, patients do not suffer from distal acroosteolysis, alopecia, severe growth failure and marked atherosclerosis. In this case, intellectual disability can be present (9–25%).

## Future challenging

One of the most attractive challenges regarding laminopathies is the search for new mutations related to specific syndromes, either in *LMNA* or related genes. On the other hand, the knowledge of their molecular basis will allow, in the future, to find specific therapies to light on what until now are life threatening and, sometimes, fatal diseases.
